# Supplementary material for: Placental Infection Associated with SARS-CoV-2 Wildtype Variant and Variants of Concern
Source: Viruses. 2023 Sep 13;15(9):1918. doi: 10.3390/v15091918 (PMC10536606; doi:10.3390/v15091918)
Supplement: Supplementary file 1 [file viruses-15-01918-s001.zip › viruses-2580737-supplementary.pdf]

Supplementary Figure S1:

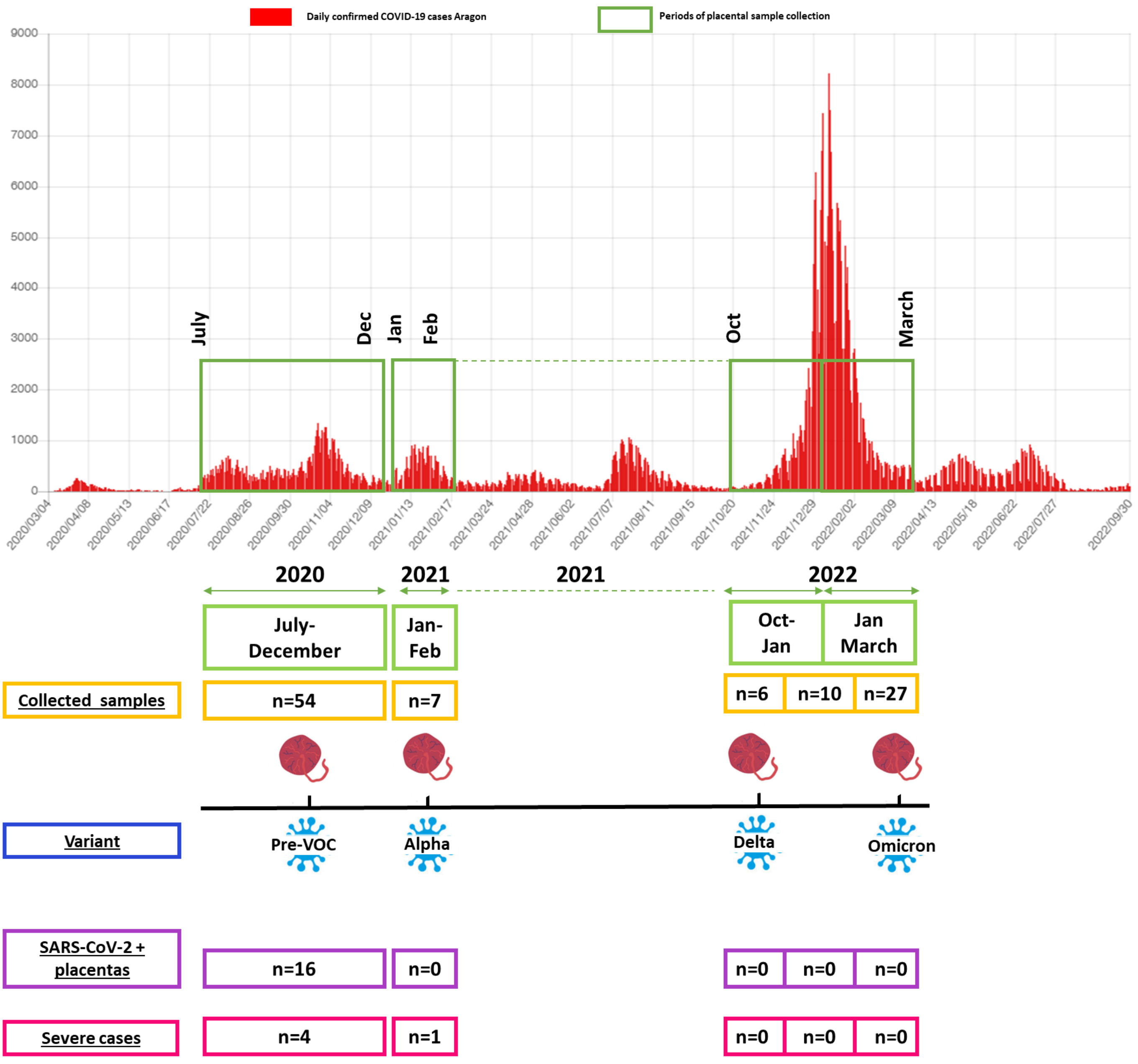

Supplementary Figure S1: Description of the SARS-CoV-2 pandemic in Aragón (Spain) and the placental samples collected in the timeframes indicated.

Supplementary Figure S2:

A

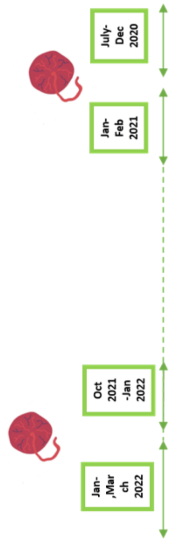

Variant, OMS designation	Lineage	Description
Pre-VOC	B.1.177	First detected in February 2020
Alpha	B.1.1.7	First detected in September 2020. Designated by OMS on 18 December 2020
Beta	B.1.351	First detected in May 2020. Designated by OMS on 18 December 2020
Gamma	P.1	First detected in November 2020. Designated by OMS on 11 January 2021
Delta	B.1.617.2	First detected in October 2020. Designated by OMS on 4 April 2021
Omicron	B.1.1.529	First detected in November 2021. Designated by OMS on 24 November 2021
Lambda	C.37	First detected in December 2020. Designated by OMS on 11 June 2021
Mu	B.1.621	First detected in January 2021. Designated by OMS on 30 August 2021

B

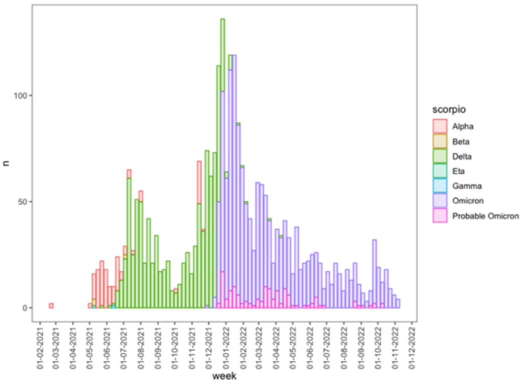

Supplementary Figure S2: A) SARS-CoV-2 variants. History of principal designated SARS-CoV-2 variants over time as designated by WHO (https://www.who.int/activities/tracking-SARS-CoV-2-variants) during preVOC (March-December 2020) and VOC era (January 2021-present). The period in which samples for this study were taken is indicated on the left. B) The ongoing evolution of variants of concern and interest of SARS-CoV-2 in Aragón, Spain. Number of samples subjected to sequencing to identify the variant/lineage of SARS-CoV-2 by time period (January 2021-present) in our area. Note that the number of samples sequenced to identify the SARS-CoV-2 variant is not representative of the number of infections reported.

Supplementary Figure S3.

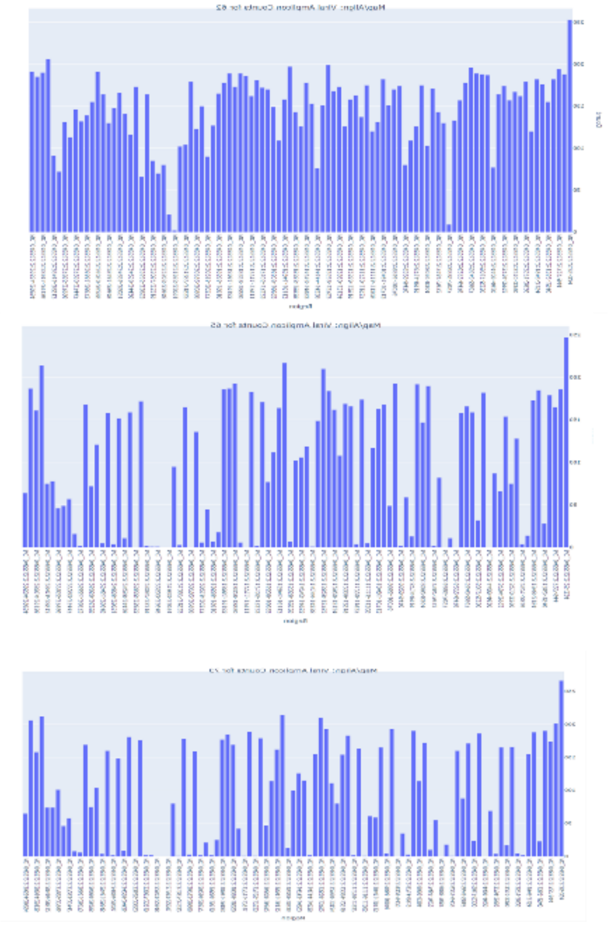

Supplementary Figure S3: Representation of coverage of the SARS-CoV-2 genome obtained by whole genome sequencing. Three representative images are shown.

FIRST DOSE	SECOND DOSE		SARS-CoV-2 INFECTION		
**Trimester**	**Days after 1º dose**	**Trimester**	**Days between last dose and SARS-CoV-2 diagnosis**	**SYN**	**PS**
3º	21	3º	61	A	Omicron
Preconception	21	Preconception	59	A	Delta
1º	-	-	142	A	Delta/Omicron
1º	-	-	133	A	Delta/Omicron
1º	21	1º	235	M	Delta/Omicron
2º	22	2º	136	A	Delta/Omicron
1º	21	1º	159	M	Delta/Omicron
2º	28	2º	100	M	Delta/Omicron
Preconception	21	1º	325	A	Omicron
2º	-	-	131	A	Omicron
Preconception	-	-	135	M	Omicron
3º	28	3º	3	M	Omicron
Preconception	344	3º	22	A	Omicron
3º	-	-	28	M	Omicron
2º	21	2º	134	A	Omicron
Preconception*#	92	Preconception	7	A	Omicron
Preconception	21	Preconception	177	M	Omicron
Preconception	291	3º	41	A	Omicron
Preconception	21	Preconception	181	M	Omicron
2º	21	2º	112	A	Omicron
2º	28	2º	104	A	Omicron
2º	21	2º	120	M	Omicron
Preconception	21	Preconception	195	M	Omicron
2º	21	2º	96	A	Omicron
Preconception*	-	-	354	A	Omicron
2º	21	2º	103	A	Omicron
1º	21	1º	195	A	Omicron

Supplementary Table S1: Temporal trends in COVID-19 vaccine in our 27 vaccinated women. Description of vaccination received by study subjects, the number of doses, the interval between dose, and timing between last dose and SARS-CoV-2 diagnosis. SYN, symptoms of SARS-CoV-2 infection; S, severe; M, mild; A, asymptomatic. PS, Predominant strain (in Aragón) at the moment of SARS-CoV-2 diagnosis. All subjects received mRNA-based vaccines except for study subjects marked*, who received Viral vector-based vaccines. Only the subject marked # received a third dose: 290 days after the first dose, during the 2º trimester of pregnancy mRNA.
